# Development and verification of a manganese metabolism- and immune-related genes signature for prediction of prognosis and immune landscape in gastric cancer

**DOI:** 10.3389/fimmu.2024.1377472

**Published:** 2024-05-13

**Authors:** Xiaoxi Han, Chuanyu Leng, Shufen Zhao, Shasha Wang, Shuming Chen, Shibo Wang, Mengqi Zhang, Xiangxue Li, Yangyang Lu, Bing Wang, Weiwei Qi

**Affiliations:** ^1^ Department of Oncology, The Affiliated Hospital of Qingdao University, Qingdao, China; ^2^ Biomedical Centre, Qingdao University, Qingdao, China

**Keywords:** gastric cancer, manganese metabolism, immune, prognostic model, immunotherapy

## Abstract

**Background:**

Gastric cancer (GC) poses a global health challenge due to its widespread prevalence and unfavorable prognosis. Although immunotherapy has shown promise in clinical settings, its efficacy remains limited to a minority of GC patients. Manganese, recognized for its role in the body’s anti-tumor immune response, has the potential to enhance the effectiveness of tumor treatment when combined with immune checkpoint inhibitors.

**Methods:**

Gene Expression Omnibus (GEO) and The Cancer Genome Atlas (TCGA) databases was utilized to obtain transcriptome information and clinical data for GC. Unsupervised clustering was employed to stratify samples into distinct subtypes. Manganese metabolism- and immune-related genes (MIRGs) were identified in GC by univariate Cox regression and least absolute shrinkage and selection operator (LASSO) regression analysis. We conducted gene set variation analysis, and assessed the immune landscape, drug sensitivity, immunotherapy efficacy, and somatic mutations. The underlying role of *NPR3* in GC was further analyzed in the single-cell RNA sequencing data and cellular experiments.

**Results:**

GC patients were classified into four subtypes characterized by significantly different prognoses and tumor microenvironments. Thirteen genes were identified and established as MIRGs, demonstrating exceptional predictive effectiveness in GC patients. Distinct enrichment patterns of molecular functions and pathways were observed among various risk subgroups. Immune infiltration analysis revealed a significantly greater abundance of macrophages and monocytes in the high-risk group. Drug sensitivity analysis identified effective drugs for patients, while patients in the low-risk group could potentially benefit from immunotherapy. *NPR3* expression was significantly downregulated in GC tissues. Single-cell RNA sequencing analysis indicated that the expression of *NPR3* was distributed in endothelial cells. Cellular experiments demonstrated that *NPR3* facilitated the proliferation of GC cells.

**Conclusion:**

This is the first study to utilize manganese metabolism- and immune-related genes to identify the prognostic MIRGs for GC. The MIRGs not only reliably predicted the clinical outcome of GC patients but also hold the potential to guide future immunotherapy interventions for these patients.

## 1 Introduction

Gastric cancer (GC), arising from the stomach’s epithelial cells, is marked by its complexity and global impact (1). As per Global Cancer Statistics 2020 (GLOBOCAN 2020), it ranks fifth in prevalence and fourth in lethality worldwide (1, 2). Early-stage gastric cancer can be treated with endoscopic techniques or surgery, but its stealthy early signs often lead to late-stage diagnosis (3). Advanced cases rely on a comprehensive approach involving systemic anti-tumor therapies, such as chemotherapy, radiation, molecular targeted therapy, and combination treatments (4, 5). However, spatial and temporal heterogeneity in gastric cancer, along with challenges from the tumor microenvironment, pose obstacles like drug resistance, limited efficacy, and tumor recurrence (6).

The immunosuppressive protein programmed death receptor 1 (PD-1) (7), which is abundant in tumor-infiltrating lymphocytes (TILs), has been shown to prevent autoimmune diseases caused by excessive immune cell activation (7, 8). The programmed death receptor-ligand 1 (PD-L1) is abundantly expressed on the cell membrane of tumor cells, and its interaction with PD-1 leads to lymphocyte death, suppression of T-cell activation, and reduction in cytokines production, ultimately enabling tumor cells to evade immune (9). Immunotherapy, especially through PD-1/PD-L1 inhibitors, exploits this characteristic of malignant cells to revive immune cells within the tumor microenvironment, reinstating the killing ability of T cells against cancer cells (10).

In recent years, monoclonal antibodies targeting PD-1/PD-L1 have been employed in clinical therapy, demonstrating effectiveness across various malignant tumors. These antibodies have notably exhibited anti-tumor effects and hold promise in extending overall survival (11–13). Unfortunately, the effectiveness of immune checkpoint inhibitors (ICIs) is hindered by the insufficient presence of immune cells within tumor microenvironments. ICIs rely on abundant immune cells to reactivate immune responses and induce anti-tumor effects (14, 15). Accordingly, the efficacy of immunotherapy is restricted to few tumor subtypes (15, 16).

Numerous metals, including potassium, calcium, manganese, and others, have been identified to modulate the immune system (17). In organisms, manganese is abundantly distributed, usually as Mn^2+^ (18). Manganese, as indispensable components in living organisms (19, 20), have been revealed to regulate cellular biological behavior, such as gene expression (21, 22) and signal transduction (23–25). During the immune surveillance process, the DNA of tumor cells activates antigen-presenting cells, particularly dendritic cells (26), leading to the synthesis of type I interferons (I-IFNs) and the presentation of tumor antigens to T cells (27, 28). This activation triggers cytotoxic T lymphocytes (CTLs) to eliminate tumor cells (26, 28), with the cGAS-STING pathway playing a significant role in this process (26, 29–31). Manganese enhances the activity of cyclic GMP-AMP synthase (cGAS) and its downstream stimulator of interferon gene (STING), resulting in a substantial increase in type I interferon production. Manganese is a potent activator of the cGAS-STING pathway (32–35), leading to noteworthy anticancer effects. Mice deficient in manganese exhibit a significant reduction in their anti-tumor potential. Additionally, combining manganese with immune checkpoint inhibitors or chemotherapeutic drugs has shown enhanced effectiveness across various tumor types (36). Consequently, the utilization of manganese in conjunction with ICIs for therapeutic interventions in malignancies holds significant promise.

This study sought to establish a prognostic signature for gastric cancer by combining immune-related genes (IRGs) and manganese metabolism-related genes (MRGs). We evaluated the significance of this prognostic model in relation to immunological characteristics, the efficacy of immunotherapy, somatic mutation, and drug sensitivity. In summary, our prognostic model holds the potential to identify new targets and treatments for gastric cancer, thereby contributing to more precise anti-tumor therapy.

## 2 Methods

### 2.1 Datasets

**Figure 1:**
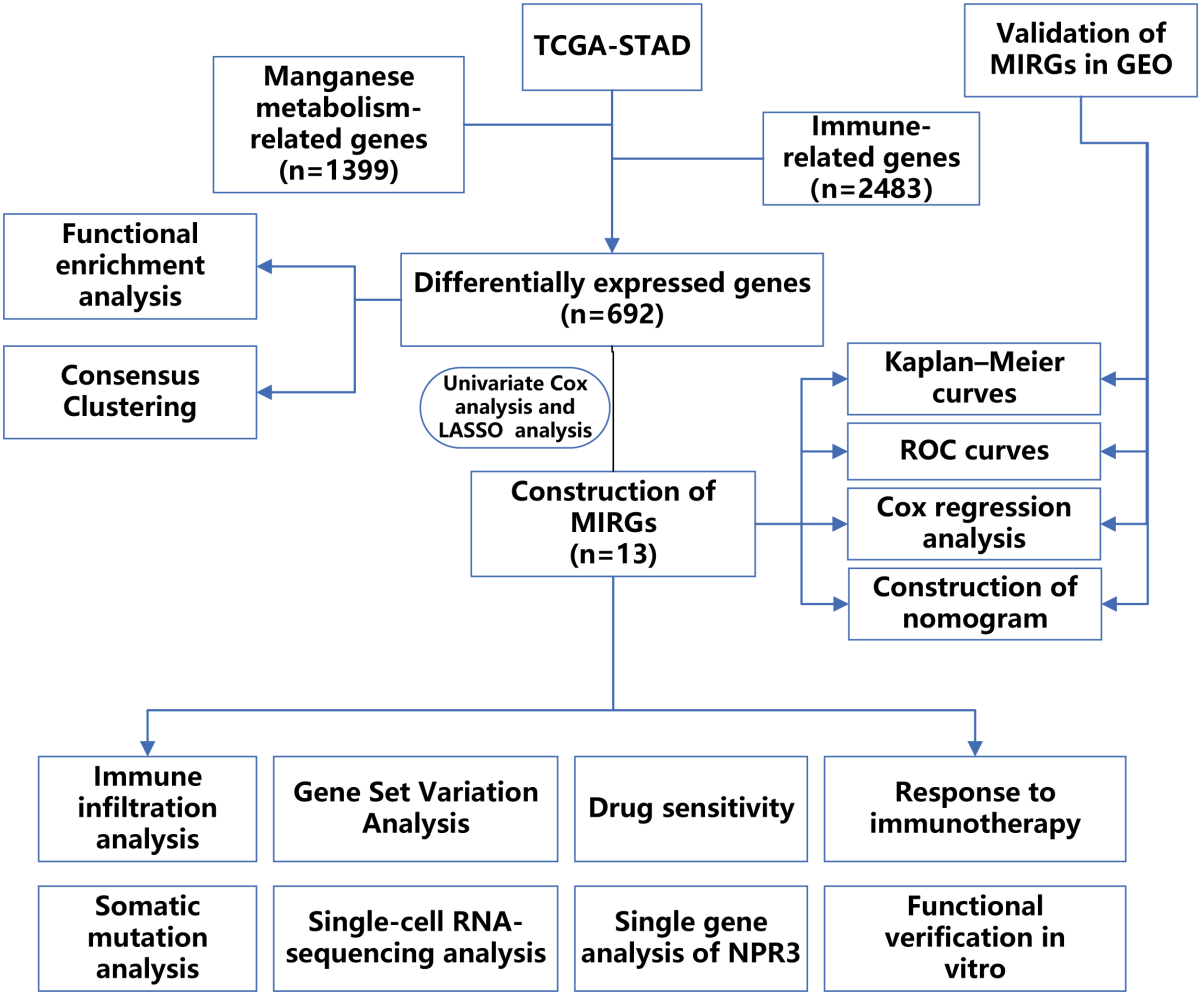
The flow chart displaying the entire research.

The RNA sequencing profiles and associated clinical data of GC were retrieved from The Cancer Genome Atlas (TCGA) and Gene Expression Omnibus (GEO) datasets. The TCGA-STAD dataset was utilized as a training cohort and was obtained from UCSC XENA (https://xenabrowser.net/datapages/). The GSE66229 dataset was used as a validation cohort and was acquired from The Gene Expression Omnibus (http://www.ncbi.nlm.nih.gov/geo/). We employed the “sva” packages to eliminate batch effects in both the TCGA-STAD dataset and the GSE66229 datasets. To ensure data integrity and precision, databases employ systematic procedures to eliminate duplicate samples and samples lacking survival data. Following the exclusion of patients with incomplete clinical information, the TCGA and GEO datasets comprised 315 and 298 GC samples, respectively. The clinical features of the samples involved in our research are displayed in **Table 1**. In addition, 1399 genes associated with manganese metabolism, referred to as manganese metabolism-related genes, were retrieved from the GeneCards database (https://www.genecards.org/) (**Supplementary Table S1**). Immune-related genes, comprising 2483 genes associated with immunity, were retrieved through the ImmPort database (https://www.immport.org/) (**Supplementary Table S2**). The flowchart depicting the procedures used in the present study is presented in **Figure 1**.

**Table 1:** Clinical characteristics of GC patients in the TCGA and GEO dataset.

Characteristic	TCGA cohort	GEO cohort
No. of patients	315	298
Status (%)		
Alive	186 (59.0%)	146 (49.0%)
Dead	129 (41.0%)	152 (51.0%)
Age (%)		
≤60	–	117 (39.3%)
>60	–	181 (60.7%)
Gender (%)		
Male	199 (63.2%)	197 (66.1%)
Female	116 (36.8%)	101 (33.9%)
WHO-Stage (%)		
I	42 (13.3%)	30 (10.1%)
II	103 (32.7%)	96 (32.2%)
II	136 (43.2%)	95 (31.9%)
IV	34 (10.8%)	77 (25.8%)
AJCC-T stage (%)		
T1	15 (4.8%)	–
T2	65 (20.6%)	186 (62.4%)
T3	150 (47.6%)	91 (30.5%)
T4	85 (27.0%)	21 (7.1%)
AJCC-N stage (%)		
N0	98 (31.1%)	38 (12.8%)
N1	84 (26.7%)	130 (43.6%)
N2	67 (21.3%)	79 (26.5%)
N3	66 (20.9%)	51 (17.1%)
AJCC-M stage (%)		
M0	294 (93.3%)	271 (90.9%)
M1	21 (6.7%)	27 (9.1%)

### 2.2 Screening of differentially expressed manganese metabolism- and immune-related genes

The raw counts of RNA-sequencing data in TCGA dataset were analyzed using the “edgeR” R package to identify differentially expressed genes (DEGs) in GC tumor tissues and normal tissues. DEGs were selected based on an absolute log2 fold change (|log2FC|) > 1 and a significance threshold of *P* < 0.05. We performed additional screening of immune- and manganese metabolism-related DEGs.

### 2.3 Functional enrichment analysis

Kyoto Encyclopedia of Genes and Genomes (KEGG) and Gene ontology (GO) pathway enrichment analysis were carried out on immune- and manganese metabolism-related DEGs applying the “clusterProfiler” package.

### 2.4 Unsupervised clustering based on immune- and manganese metabolism-related DEGs

The process of consistent clustering was carried out on immune-related and manganese metabolism-related DEGs using the “NMF” R package. The subtypes were then subjected to a t-distributed stochastic neighbor embedding (t-SNE) analysis. The analysis of survival differences among subtypes was conducted utilizing the “survminer” and “survival” R packages. We investigated the variations in Estimation of STromal and Immune cells in MAlignant Tumor tissues using Expression data (ESTIMATE) score and tumor purity among different subtypes. Additionally, the comparative analysis of immune checkpoint expression across various subgroups was assessed by Wilcoxon test. Gene Set Variation Analysis (GSVA) was applied to investigate the differences in enrichment pathways across various subtypes.

### 2.5 Construction and verification of MIRGs based on manganese metabolism and immune characteristics

Data from TCGA was analyzed using univariate Cox regression analysis to evaluate the predictive significance of DEGs associated with manganese metabolism and immune on overall survival (OS), with a threshold of *P* < 0.01. A least absolute shrinkage and selection operator (LASSO) regression analysis was carried out using the R package “glmnet” to avoid the issue of overfitting and develop a prognostic manganese metabolism- and immune-related genes (MIRGs)-based signature. The risk scores for every sample in the training and validation set were derived via a specific formula: Risk score = ∑^n^_i_ coefi * Expression (X_i_). The term “Coefi” represented the coefficient value associated with each gene, and “Expression (Xi)” represented each gene’s expression value. By employing the median risk score as the criterion for division, the patients within both the training and validation datasets were categorized into high-risk and low-risk cohorts. The prognostic differences across subgroups were assessed using the log-rank test and Kaplan-Meier survival analysis, implemented in the “survminer” and “survival” R packages.

To evaluate the prediction accuracy and effectiveness of MIRGs, the receiver operating characteristic (ROC) curve, and time-dependent ROC (timeROC) curve were generated using the “survivalROC” and “timeROC” R packages. The predictive performance of Manganese metabolism- and immune-related genes (MIRGs) was assessed using the area under curve (AUC). To identify independent prognostic features capable of predicting survival states in gastric cancer patients, univariate and multivariate Cox regression analyses were conducted using risk scores and clinical features. Additionally, the predictive performance of MIRGs was externally validated using the GSE66229 dataset.

### 2.6 Establishment of a nomogram

“rms” R package to construct a nomogram that integrated clinical features and risk scores, aiming to predict overall survival at 1, 2, and 3 years. ROC curves and calibration curves were generated to evaluate the predictive effectiveness of the nomogram.

### 2.7 Analysis of tumor microenvironment

Immune cell infiltration data were derived using Cell-type Identification By Estimating Relative Subsets Of RNA Transcripts (CIBERSORT) algorithms (37). Enrichment scores for immunological functions were computed using the “GSVA” and “GSEABase” packages, employing single-sample gene set enrichment analysis (ssGSEA). Subsequently, Wilcoxon test was carried out to identify differences in immune functions and immune cell profiles between individuals categorized as low-risk and high-risk.

### 2.8 Gene set variation analysis of MIRGs

The “GSVA” package was employed to conduct GSVA analysis, aiming to investigate the molecular functions and signaling pathways associated with MIRGs.

### 2.9 Prediction of drug sensitivity

To provide management recommendations for GC, we analyzed the half-maximal inhibitory concentration (IC_50_) of 198 drugs sourced from the Genomics of Drug Sensitivity in Cancer (GDSC) database. The investigation utilized the “oncoPredict” R package. Additionally, Wilcoxon test was used to compare the sensitivity differences of commonly used medications in the clinical treatment of GC between the low-risk group and high-risk group. Moreover, Spearman correlation test was performed to examine the relationship between the IC_50_ values of commonly used drugs and risk scores.

### 2.10 Responses to immunotherapy

Microsatellite instability (MSI) is characterized by a high mutation rate due to single nucleotide substitutions and frequent variations in short repetitive DNA sequences (38), resulting from a failure in DNA mismatch repair (MMR) (38). Subsequently, an examination was conducted to assess the mutational status of microsatellites in patients diagnosed with gastric cancer. Additionally, an investigation explored the correlation between the mutation status of microsatellites and the corresponding risk scores. The Tumor Immune Dysfunction and Exclusion (TIDE) score for each sample was obtained using the “TIDE” method, predicting immunotherapy efficacy, where a lower TIDE score indicates a more positive immunotherapeutic effectiveness (39). The immunophenoscore (IPS) of patients from TCGA-STAD was derived through The Cancer Immunome Atlas (TCIA) website (https://tcia.at), enabling the prediction of the effect of immune checkpoint inhibitors such as CTLA4 and PD-1/PD-L1 antagonists (40). The IPS score underwent normalization, resulting in a scale ranging from 0 to 10, with a higher IPS score implying an elevated level of immunological reactivity. Furthermore, an analysis was performed on the differential expressions of immunological checkpoints among various risk groups. The Wilcoxon test was used to determine if there are statistically significant differences between two groups.

### 2.11 Tumor mutation burden analysis

Tumor mutational burden (TMB) quantifies the number of mutated bases per million bases in each tumor sample, encompassing various mutation types like missense mutations, frameshift mutations, and nonsense mutations. The TMB data for stomach adenocarcinoma were extracted from TCGA and can be accessed at https://portal.gdc.cancer.gov/. The somatic point mutations in each gastric cancer sample were visualized by generating waterfall charts using the “maftools” R package.

### 2.12 Single-cell RNA-sequencing analysis

The single-cell RNA sequencing (scRNA-seq) dataset GSE184198 was obtained from GEO for further investigation. The “Seurat” R program was employed to analyze this dataset, which includes both gastric cancer and normal tissue samples. To ensure high-quality scRNA-seq data, an initial filtering process was applied to retain cells expressing a minimum of 200 genes and genes exhibiting expression in at least three cells. Additionally, cells with mitochondrial gene expression exceeding 5% were excluded. Subsequently, the scRNA-seq data were normalized, and 2000 genes with significant variability were identified. Principal component analysis (PCA) was utilized to assess the significance of principal components between tissues or cells, and the datasets were visualized using uniform manifold approximation and projection (UMAP). CellMarker 2.0 was used to annotate cells in each cluster. Differentially expressed genes for each cluster were identified using a threshold of |log2FC| > 0.25. Furthermore, the distribution of the *NPR3* gene within the cell clusters was explored.

### 2.13 Single gene analysis of *NPR3*

The *NPR3* gene, selected from the prognostic model, underwent additional analysis. The *NPR3* expression levels in normal tissues and gastric cancer were compared using the Wilcoxon test. Subsequently, ROC curve analysis and Kaplan-Meier survival analysis were performed to illustrate the prognostic significance of *NPR3*. Furthermore, an exploration of the correlation between different immune cells and *NPR3* was conducted.

### 2.14 Cell and reagents

The following cell lines, antibodies, and chemicals were employed in this research: GES-1, AGS, MKN7, SGC7901, NCI-N87 (Cell Bank of Type Culture Collection of the Chinese Academy of Sciences), anti-NPR3 (Abcam, A19038), anti-GAPDH (Proteintech, Cat No: 10494–1-AP), anti-E-cadherin (Proteintech, Cat No: 60335–1-Ig), anti-N-cadherin (Proteintech, Cat No: 22018–1-AP), anti-Vimentin (Proteintech, Cat No: 10366–1-AP), HRP Goat Anti Rabbit IgG (H+L) (Abclonal, AS014), Crystal violet (Aladdin, C110703), 3-(4,5-dimethylthiazolyl-2)-2,5-diphenyltetrazolium bromide (MTT, Aladdin: M158055) and BeyoClick ™ EdU-488 Cell Proliferation Detection Kit (Beyotime, C0071S).

### 2.15 Establishing stably transfected cell lines

Jikai Gene (Shanghai, China) supplied the lentivirus (vector: GV341) used in the study. Initially, AGS and SGC7901 cells were inoculated in a 6-well plate and allowed to stabilize for 24 hours. Following this, the lentivirus was added to the culture medium, and the cells were incubated for an additional 24 hours. Subsequently, puromycin was applied to the cells for 48 hours, following the provided instructions, to identify cell lines that stably overexpress *NPR3* (p-NPR3).

### 2.16 Cell viability determination

AGS (1.5*10^4^/well) and SGC7901 (3*10^4^/well) cells were seeded into a 24-well plate. Cell viability was assessed on the first, third, and fifth days. At each time point, cell growth was halted, and a 0.5 mg/mL MTT solution was introduced to the 24-well plate, followed by 2–4 hours of incubation. Subsequently, the resulting solution was dissolved in DMSO, and the absorbance was measured at 490 nm using a full-function microplate detector (BioTek, USA).

### 2.17 EdU assay

AGS and SGC7901 cells were cultured in a 24-well plate. The EdU working solution was introduced to the cell culture system when the cells were in a healthy state and they were allowed to incubate for 2 hours. Subsequently, in accordance with the provided instructions, the EDU reaction solution and DAPI were added separately. The scene was observed and photographed using an inverted fluorescence microscope (Nikon, Japan).

### 2.18 Migration assay

AGS and SGC7901 cells were inoculated for growth in a 24-well plate. Once each well was filled with cells, a wound was created on the cell surface using a tip. To assess the migratory ability of the cells, the width of the wound was measured with a microscope at 0 and 48 hours.

### 2.19 Colony formation assay

AGS (1000/well) and SGC7901 (1000/well) cells were inoculated in a 6-well plate, and the culture media were changed every three days. The cells were fixed with 4% paraformaldehyde, stained with a 0.1% crystal violet solution, and washed with tap water until the 12th day. Subsequently, the cell colonies were examined, and the count was conducted.

### 2.20 Western blot

To obtain cell proteins, cells in good growth condition were collected and lysed on ice for 1 hour in cell lysis buffer for Western and IP. Cell proteins were quantified using the BCA method, and a buffer solution was added to achieve 3000μg/ml. The protein was denatured by boiling in water at 100°C for 10 minutes. Subsequently, cell proteins were subjected to electrophoresis at 80V and 120V, followed by protein transfer to polyvinylidene difluoride (PVDF) membranes at 300mA for 120 minutes.

The PVDF membrane was sealed at room temperature for 2 hours, then incubated overnight with anti-NPR3 and anti-GAPDH. Antibodies were diluted at a ratio of 1:1000 using 5% skim milk. Following this, HRP Goat Anti-Rabbit IgG (H+L) was left at room temperature for 2 hours before testing for protein expression.

### 2.21 Statistical analysis

The statistical analyses were conducted using R software (version 4.3.0) and GraphPad Prism Software (version 8.0.2). The independent Student’s t-test was performed to compare normally distributed continuous data between two groups, while the Wilcoxon test was used to compare non-normally distributed continuous variables. Kruskal-Wallis test was used for comparison of continuous variables between multiple groups. The Spearman correlation test was used to measure the correlation between two variables. Experimental data were presented as means ± standard deviation (SD). *P* < 0.05 were deemed statistically significant unless otherwise stated in the text.

## 3 Results

### 3.1 Screening of differentially expressed manganese metabolism- and immune-related genes

**Figure 2:**
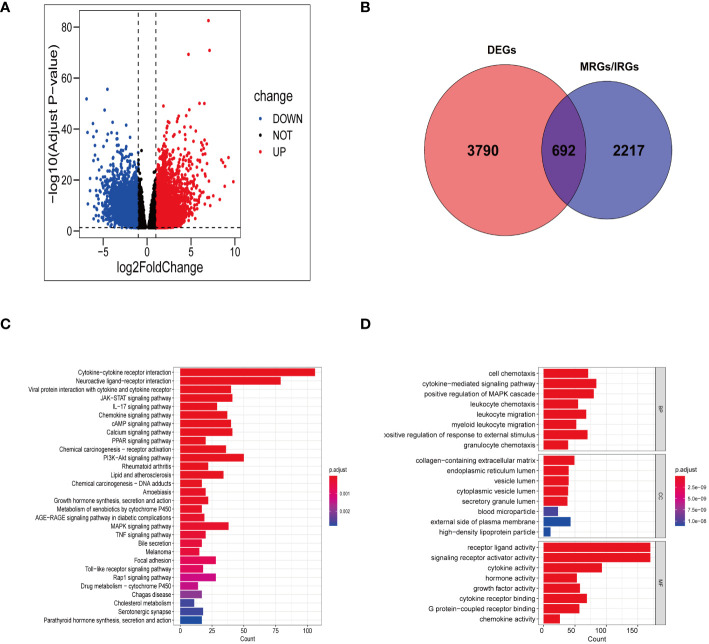
Gene screening and functional enrichment analysis. **(A)** Volcanic plot of DEGs in TCGA-STAD. **(B)** Venn diagram showing 692 differentially expressed MRGs and IRGs. **(C, D)** Bar chart revealing the outcomes of KEGG pathways enrichment **(C)** and GO functional enrichment **(D)** of 692 differentially expressed MRGs and IRGs.

In TCGA-STAD, a total of 4482 genes displayed differential expression between normal tissues and gastric tumor samples (**Supplementary Table S3**). Among these genes, 2133 were upregulated, while 2349 were downregulated (**Figure 2A**). Our analysis involved a comprehensive set of 2909 genes related to manganese metabolism and immune functions (MRGs and IRGs). From this pool, 692 genes were identified as DEGs associated with both manganese metabolism and immune functions (**Figure 2B**; **Supplementary Table S4**).

### 3.2 The evaluation of functional analysis

The KEGG functional annotation revealed enrichment in immune activation pathways, including cytokine-cytokine receptor interaction, IL-17 signaling pathway, viral protein interaction with cytokine and cytokine receptor, Toll-like receptor signaling pathway, chemokine signaling pathway, and neuroactive ligand-receptor interaction (**Figure 2C**; **Supplementary Table S5**). The bar plot (**Figure 2D**) displayed the top 8 results in Biological Process (BP), Molecular Function (MF), and Cellular Component (CC) (**Supplementary Table S6**). The GO functional annotation analysis indicated that the DEGs associated with manganese metabolism and immune functions primarily play roles in cell chemotaxis, cytokine receptor binding, granulocyte chemotaxis, leukocyte migration, and cytokine-mediated signaling pathways.

### 3.3 Construction of the four subtypes by unsupervised clustering

**Figure 3:**
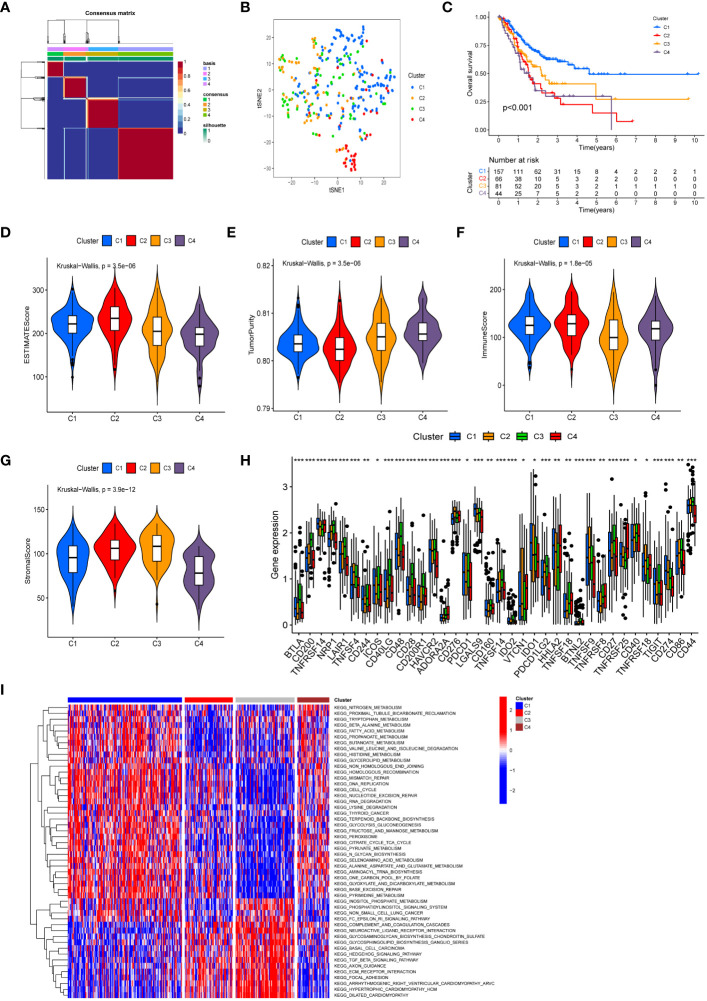
Molecular subtypes based on differentially expressed MRGs and IRGs. **(A)** Heatmap of consensus clustering matrix (k=4) showing four clusters (C1 = 157; C2 = 66; C3 = 81; C4 = 44) for MIRGs. **(B)** tSNE plot depicting distribution of four clusters. **(C)** The Kaplan–Meier curves displaying significant differentiation in overall survival time of patients between different phenotypes (*P* < 0.001). **(D–G)** Violin plots showing the ESTIMATE score **(D)**, tumor purity **(E)**, immune score **(F)** and stromal score **(G)** across different phenotypes. **(H)** The box diagrams displaying the difference of checkpoints’ expression in four clusters. **(I)** Heatmap of GSVA demonstrating biological functions and signaling pathways in four subgroups. (*, **, *** represent P < 0.05, P < 0.01, P < 0.001, respectively.).

Following cluster analysis based on manganese metabolism- and immune-associated DEGs, the entire cohort was stratified into four distinct subtypes, denoted as clusters 1, 2, 3, and 4 (**Figure 3A**). The t-SNE analysis results are presented in **Figure 3B**. Furthermore, we conducted an analysis of prognostic differences among the four subtypes. As illustrated in **Figure 3C**, there was a statistically significant difference in prognosis among the four subgroups (*P* < 0.001). Cluster 1 exhibited longer overall survival times compared to the other clusters, predicting a more favorable prognosis for cluster 1. The variations in the tumor microenvironment among distinct subtypes were demonstrated in **Figures 3D–G**. Cluster 2 showed significantly increased immunological scores and ESTIMATE scores compared to the other clusters. The tumor purity in cluster 4 was higher than in the other clusters, potentially explaining its poor prognosis. Evaluation of immunological checkpoints revealed that patients in cluster 3 had significantly elevated expression levels for most immune checkpoints compared to patients in the other subgroups (**Figure 3H**). According to the GSVA results (**Figure 3I**; **Supplementary Table S7**), signaling pathways linked to tumor progression, such as focal adhesion, the transforming growth factor-β (TGF-β) signaling pathway, and extracellular matrix (ECM) receptor interaction, were elevated in clusters 2 and 3. Metabolic pathways, including pyruvate metabolism, tryptophan metabolism, and fatty acid metabolism, were concentrated in Clusters 1 and 4.

### 3.4 Establishment and validation of MIRGs

**Figure 4:**
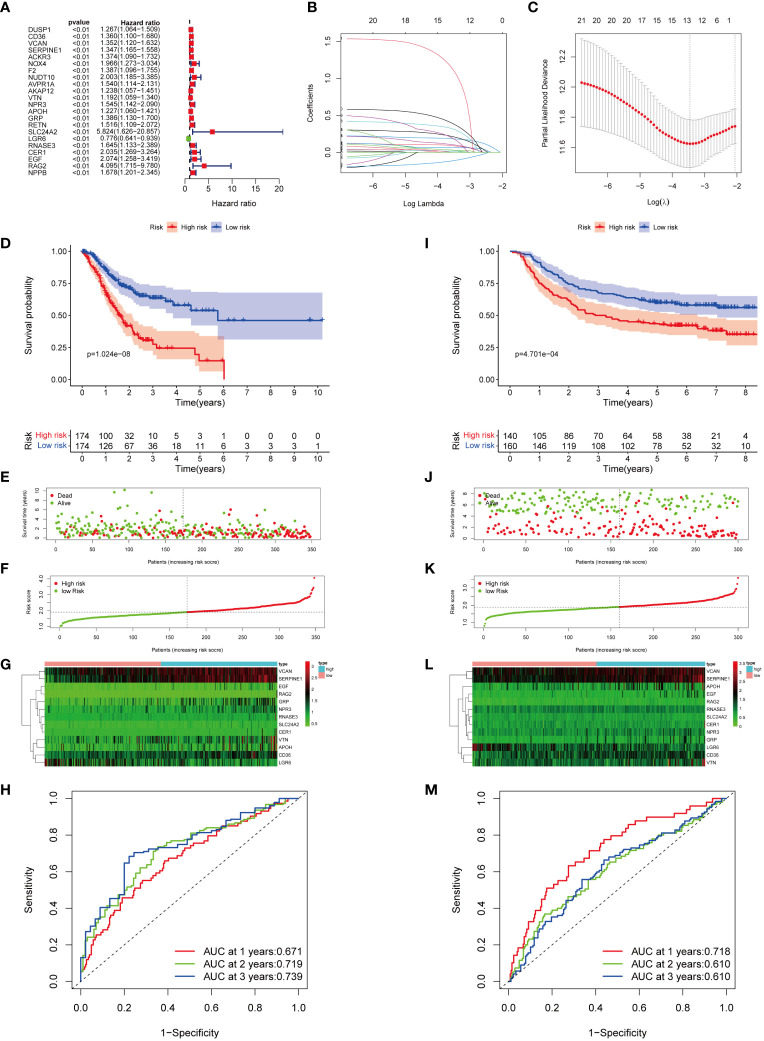
Construction and verification of MIRGs. **(A)** Forest plot of univariate cox regression analysis screening 22 DEGs linked to survival. **(B, C)** LASSO regression generating 13 genes for MIRGs. **(D)** The Kaplan-Meier curves illustrating significant differentiation in survival of patients between risk subgroups in the TCGA cohort. **(E)** Distribution of survival status in the TCGA cohort. **(F)** Distribution of risk score in the TCGA cohort. **(G)** Heatmaps of 13 genes from MIRGs in the TCGA cohort. **(H)** ROC curves evaluating the predictive accuracy of MIRGs in the TCGA cohort. **(I)** The Kaplan-Meier curves illustrating significant differentiation in survival of patients between risk subgroups in the GEO cohort. **(J)** Distribution of survival status in the GEO cohort. **(K)** Distribution of risk score in the GEO cohort. **(L)** Heatmaps of 13 genes from MIRGs in the GEO cohort. **(M)** ROC curves evaluating the predictive accuracy of MIRGs in the GEO cohort.

Univariate Cox regression analysis showed a significant correlation between 22 genes and OS in GC patients within the TCGA cohorts (**Figure 4A**; **Supplementary Table S8**). Among them, LGR6 was identified as protective for GC (HR < 1, *P* < 0.01), while the remaining genes were considered risk factors (HR > 1, *P* < 0.01). LASSO regression analysis identified 13 genes as MIRGs (**Figures 4B, C**; **Supplementary Table S9**). Regression coefficients were computed for each of the 13 genes. Among the 13 MIRGs, 5 were classified as Manganese Metabolism-Related Genes (*CD36, VCAN, SERPINE1, SLC24A2, RAG2*), 3 as Immune-Related Genes (*APOH, LGR6, CER1*), and 5 as genes belonging to both MRGs and IRGs (*VTN, NPR3, GRP, RNASE3, EGF*). A patient’s risk score was determined based on a specific formula: RiskScore = (0.086 × CD36) + (0.024 × *VCAN*) + (0.182 × *SERPINE1*) + (0.065 × *VTN*) + (0.011 × *NPR3*) + (0.099 × *APOH*) + (0.205× *GRP*) + (0.027 × *SLC24A2*) + (-0.108 × *LGR6*) + (0.304 × *RNASE3*) + (0.171 × *CER1*) + (0.372 × *EGF*) + (1.075 × *RAG2*).

The median risk score of the TCGA cohort served as the threshold value to categorize GC patients in the training set into high- and low-risk groups. Kaplan-Meier survival analysis exhibited that the high-risk group of GC patients experienced an increased mortality rate and a reduced overall survival duration (**Figure 4D**). The survival outcome and risk scores of samples in the training cohort were shown in **Figures 4E, F**. The variances in expression of the thirteen genes among subgroups was displayed in **Figure 4G**. The efficiency of MIRGs was evaluated using the time-dependent curve, with area under the curves (AUCs) of 0.671, 0.719, and 0.739 at 1, 2, and 3 years (**Figure 4H**). The risk score’s AUC was 0.657, the highest among all other clinical characteristics (**Supplementary Figure S1A**). The risk score for every sample in the GSE66229 dataset was determined using the same method as in TCGA. To validate MIRGs constructed from TCGA dataset, patients in the GSE66229 datasets were also separated into high- and low-risk subgroups using the same threshold. Similar outcomes were achieved in the validation cohort. Patients in high-risk groups had a significantly reduced survival duration compared to those in the low-risk group (P < 0.001) (**Figure 4I**). The survival status and risk scores of samples in the validation cohort were presented in **Figures 4J, K**. **Figure 4L** displayed the variances in expression of the thirteen genes between the high- and low-risk subgroups. Time-dependent ROC curve indicated favorable levels of sensitivity and specificity, with AUC values at 1, 2, and 3 years of 0.718, 0.610, and 0.610, respectively (**Figure 4M**). The risk score AUC was 0.719, reflecting that MIRGs were highly efficient in predicting the outcome of individuals with GC (**Supplementary Figure S1B**). Furthermore, the univariate and multivariate Cox regression analysis illustrated that MIRGs were significant predictors for GC in both the TCGA-STAD dataset (**Supplementary Figures S1C, D**) and the GSE66229 dataset (**Supplementary Figures S1E, F**).

### 3.5 Development of the risk score-related prognostic nomogram

**Figure 5:**
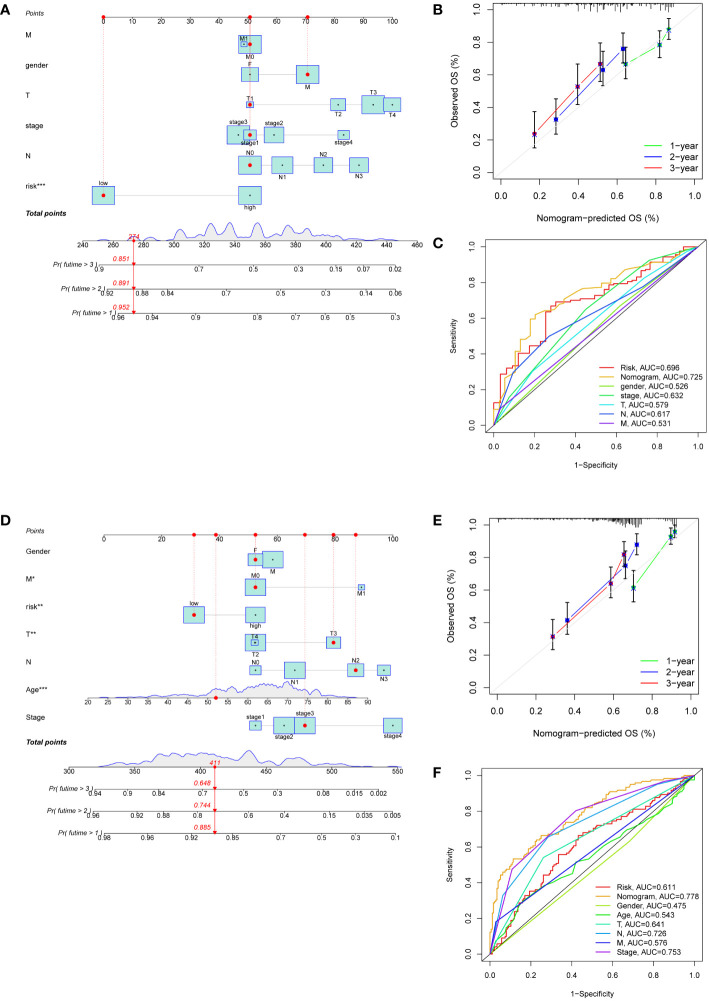
Development of nomograms based on clinical features and risk scores. **(A, D)** The nomograms predicting the 1-, 2- and 3-year survival rate of patients with GC in the TCGA cohort **(A)** and in the GEO cohort **(D)**. **(B, E)** Calibration curves for nomograms in the TCGA cohort **(B)** and in the GEO cohort **(E)**. **(C, F)** ROC curves assessing the prognostic accuracy of nomogram and other clinical features in the TCGA cohort **(C)** and in the GEO cohort **(F)**.

Nomograms were developed to enhance the predictive accuracy and clinical utility for GC patients by incorporating a risk score and other prognostic indicators (**Figures 5A, D**). The overall score and the patient survival rates at one, two, and three years showed a negative connection. Calibration curves demonstrated a close correspondence between the anticipated 1-, 2-, and 3-year rates of survival and the real-world survival rates (**Figures 5B, E**). Additionally, the AUC of the nomogram ROC curve was 0.725 in the training set and 0.778 in the validation set (**Figures 5C, F**). These values exceeded those for gender, age, risk, and TNM stage, indicating that the nomogram exhibited significant promise in predicting survival rates for GC patients.

### 3.6 Immunological features of MIRGs

**Figure 6:**
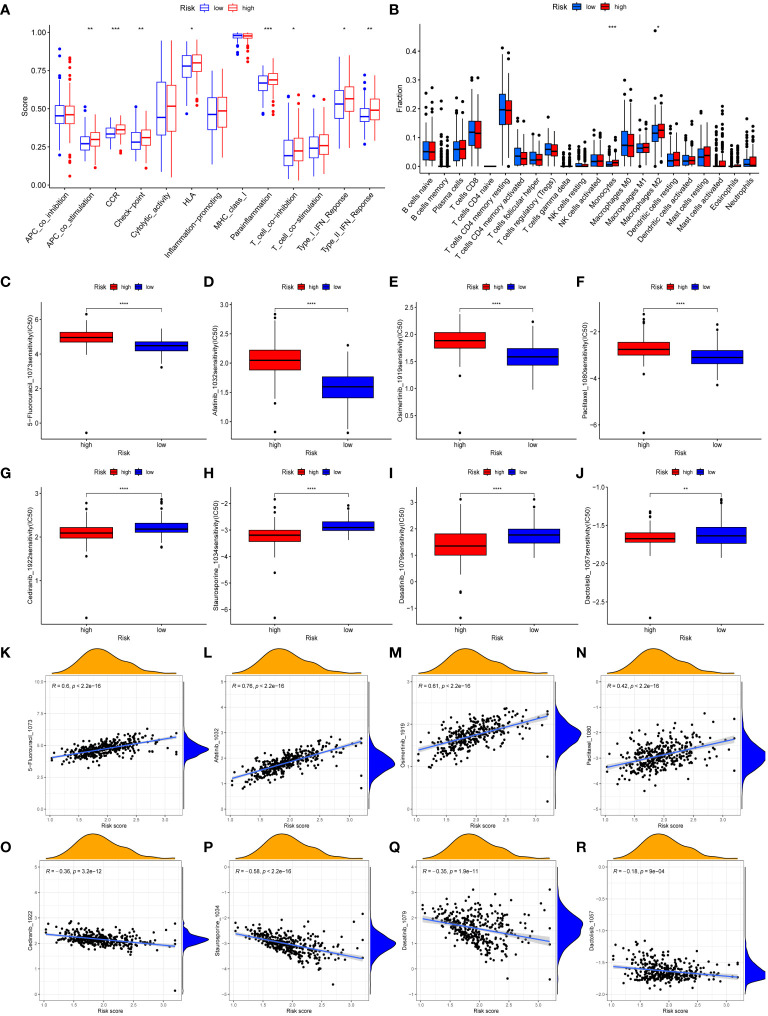
Immune landscape and drug sensitivity. **(A, B)** Evaluation of immune cell infiltration **(A)** and immune function **(B)** between risk subgroups. **(C–J)** Comparison of the IC_50_ values of 5-Fluorouracil **(C)**, afatinib **(D)**, osimertinib **(E)**, paclitaxel **(F)**, cediranib **(G)**, staurosporine **(H)**, dasatinib **(I)**, and dactolisib **(J)** between risk subgroups. **(K–R)** The correlation between IC_50_ values of 5-Fluorouracil **(K)**, afatinib **(L)**, osimertinib **(M)**, paclitaxel **(N)**, cediranib **(O)**, staurosporine **(P)**, dasatinib **(Q)**, dactolisib **(R)** and risk scores. (*, **, ***, **** represent P < 0.05, P < 0.01, P < 0.001, P < 0.0001, respectively.).

The immune-related scores in TCGA-STAD were computed using the CIBERSORT algorithm, allowing us to differentiate immune infiltration between risk subgroups. High-risk individuals exhibited a significant enhancement in many immunological functions, including APC co-stimulation, circulating chemokine-receptor (CCR), human leukocyte antigen (HLA), immune checkpoints, Parainflammation, type-I IFN response, type-II IFN response, and T cell co-inhibition (**Figure 6A**). The analysis of immune infiltration revealed that patients classified as high-risk exhibited a greater fraction of monocytes and M2 macrophages compared to patients classified as low-risk (**Figure 6B**). The immunological characteristics of distinct risk subgroups exhibited significant variation.

### 3.7 Pathway enrichment analysis of MIRGs

The purpose of conducting GSVA was to investigate the variations in the enrichment of signaling pathways or biological processes between risk subgroups (**Supplementary Figure S2A**). Patients classified as high-risk showed enrichment in various processes and pathways associated with the progress and metastasis of tumor, including focal adhesion, TGF-β signaling pathway, Janus kinase/signal transducer and activator of transcription (JAK/STAT) signaling pathway, and cell adhesion molecules (CAMs). Patients classified as low-risk exhibited enrichment in metabolic-related processes, specifically propanoate metabolism, butanoate metabolism, pyruvate metabolism, and pyrimidine metabolism. Additionally, gene repair-related pathways, which includes nucleotide excision repair, base excision repair, and mismatch repair, were enriched in the low-risk group.

### 3.8 Drug sensitivity analysis

We conducted a sensitivity analysis on 198 drugs sourced from the GDSC database (**Supplementary Table S10**). We then examined the IC_50_ values of clinically relevant medicines between the low-risk and high-risk groups (**Figures 6C–J**). Furthermore, the association between IC_50_ values of medicines and risk scores was demonstrated (**Figures 6K–R**). The IC_50_ values of 5-Fluorouracil, afatinib, osimertinib, and paclitaxel medicines exhibited an upward trend when the risk scores increased. Conversely, the IC_50_ values of cediranib, dactolisib, dasatinib, and staurosporine medicines were reduced as the risk scores increased. Drug sensitivity analysis revealed that individuals classified in the high-risk groups could potentially derive therapeutic benefits from cediranib, dactolisib, dasatinib, and staurosporine. Patients categorized as low-risk demonstrated a greater propensity to benefit from 5-Fluorouracil, afatinib, osimertinib, and paclitaxel.

### 3.9 Evaluation of immunotherapy efficacy

**Figure 7:**
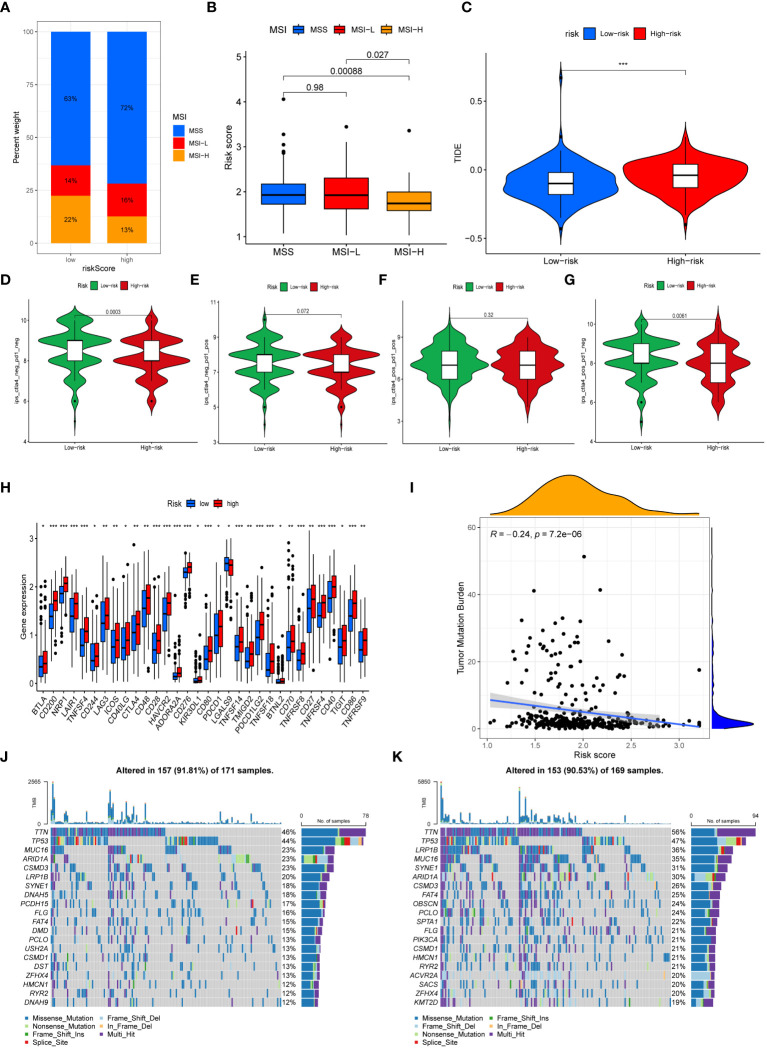
Immunotherapy efficacy and Tumor Mutational Burden. **(A)** The mutation status of microsatellites. **(B)** The difference of the median risk scores in the three subtypes. **(C)** Comparison of the TIDE score between risk subgroups. **(D–G)** The differences of Immunophenoscores (IPS) **(D)**, IPS-PD1/PD-L1/PD-L2 **(E)**, IPS-PD1/PD- L1/PD-L2+CTLA4 **(F)**, and IPS-CTLA4 **(G)** between risk subgroups. **(H)** Comparison of immune checkpoints expression between risk subgroups. **(I)** Correlation analysis of risk scores and TMB. **(J, K)** The waterfall map demonstrating mutation frequencies in high-risk group **(J)** and in low-risk group **(K)**. (*, **, *** represent P < 0.05, P < 0.01, P < 0.001, respectively.).

We next performed an analysis of the distribution of microsatellite states among patients (**Figure 7A**). The percentage of individuals with microsatellite stability (MSS) was greater in both high-risk and low-risk groups. In addition, the percentage of individuals with microsatellite instability (MSI) in low-risk patients (36%) exceeded that in the high-risk patients (29%). Individuals with MSI-high (MSI-H) status had a significantly lower risk score than those with MSS and MSI-low (MSI-L) status (**Figure 7B**). This demonstrated that immunotherapy was more likely to benefit GC patients categorized as low-risk. Patients who had higher TIDE scores often experienced less sensitivity to immunotherapy. As depicted in **Figure 7C**, patients categorized as low-risk had significantly elevated TIDE scores, suggesting that immunotherapy might have diminished efficacy. An analysis was further conducted to assess the IPS scores of GC patients in different risk subgroups (**Figures 7D–G**). We found that the IPS-CTLA4 blocker score was significantly higher in the low-risk group compared to the high-risk group. This suggested that GC patients categorized as low-risk could potentially benefit from ICIs. Moreover, the low-risk group’s immune checkpoint expression levels were lower than those of the high-risk group (**Figure 7H**).

### 3.10 Somatic mutation analysis

The TMB of each sample was computed and analyzed. The findings demonstrated that the risk score and the TMB were negatively correlated, with the high-risk group exhibiting a significantly lower TMB in contrast to the low-risk group (**Figure 7I**). Waterfall plots were generated to display the top 20 genes exhibiting the highest mutation frequencies in high-risk patients (**Figure 7J**) and low-risk patients (**Figure 7K**).

### 3.11 Single-cell RNA-sequencing data

**Figure 8:**
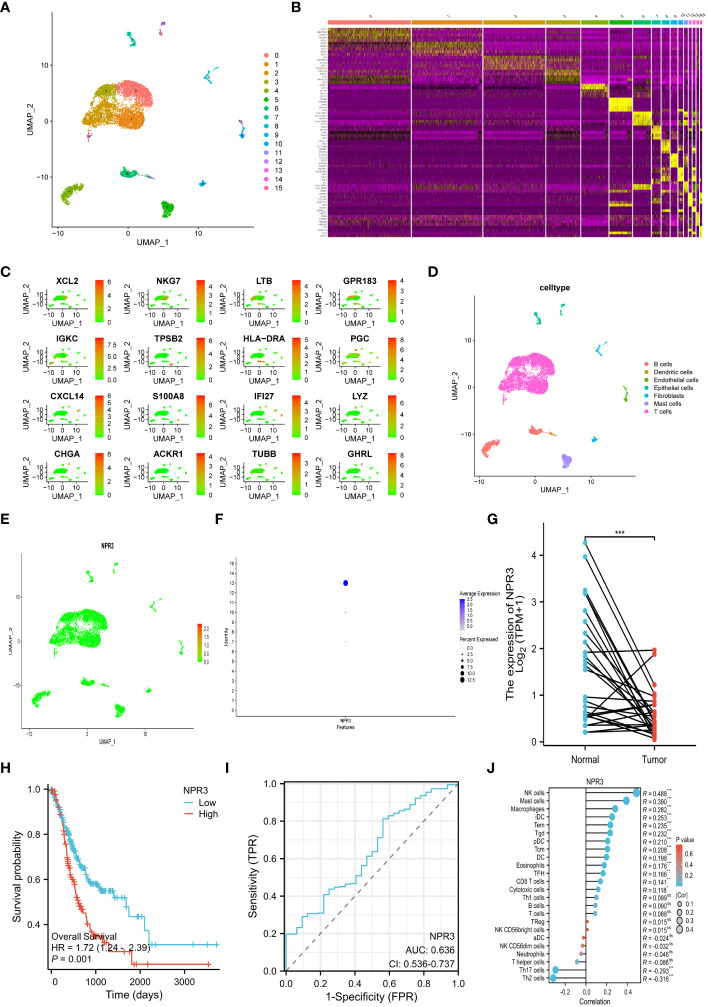
Single-cell RNA-sequencing analysis and single gene analysis of *NPR3*. **(A)** UMAP plot of 16 cell clusters. **(B)** Heatmap showing the top 5 marker genes for each cluster. **(C)** Scatter plot depicting the distribution of the marker gene for each cluster. **(D)** UMAP plot of all clusters with cell-type annotations. **(E, F)** Scatter plot **(E)** and bubble plot **(F)** displaying the distribution of *NPR3* genes in clusters. **(G)** Differential expression of *NPR3* between normal tissues and gastric cancer. **(H)** The Kaplan-Meier survival analysis of *NPR3*. **(I)** ROC curve of *NPR3* in predicting survival time. **(J)** Correlation between various immune cells and *NPR3*. (*, **, ***, and ns represent p < 0.05, p < 0.01, p < 0.001, and “not statistically”, respectively.).

The transcriptional profile of 22,240 cells were derived from the scRNA-seq data of GSE184198. Then, sixteen cell clusters were visualized by UMAP (**Figure 8A**). A heatmap was generated to display the expression of the top 5 marker genes for each cluster (**Figure 8B**). We also visualized the distribution of the marker gene for each cluster in the scatter plot (**Figure 8C**). The sixteen cell clusters could be divided into seven distinct categories of cell types: T cells, dendritic cells, B cells, epithelial cells, fibroblasts, endothelial cells, and mast cells (**Figure 8D**). Furthermore, the expression and distribution of the *NPR3* gene were visualized (**Figures 8E, F**). The data indicated that *NPR3* was only enriched in endothelial cells.

### 3.12 Single gene analysis of *NPR3*

The *NPR3* gene exhibited significantly decreased expression in GC tissues (**Figure 8G**). Individuals with high *NPR3* expression had a worse prognosis compared to those with low *NPR3* expression (**Figure 8H**). The AUC of the ROC curve was 0.636, indicating that the *NPR3* gene provided accurate prognostic prediction (**Figure 8I**). The *NPR3* gene is linked to a diverse range of immune cells, particularly mast cells, macrophages, and NK cells (**Figure 8J**).

### 3.13 Function validation of *NPR3* in GC

**Figure 9:**
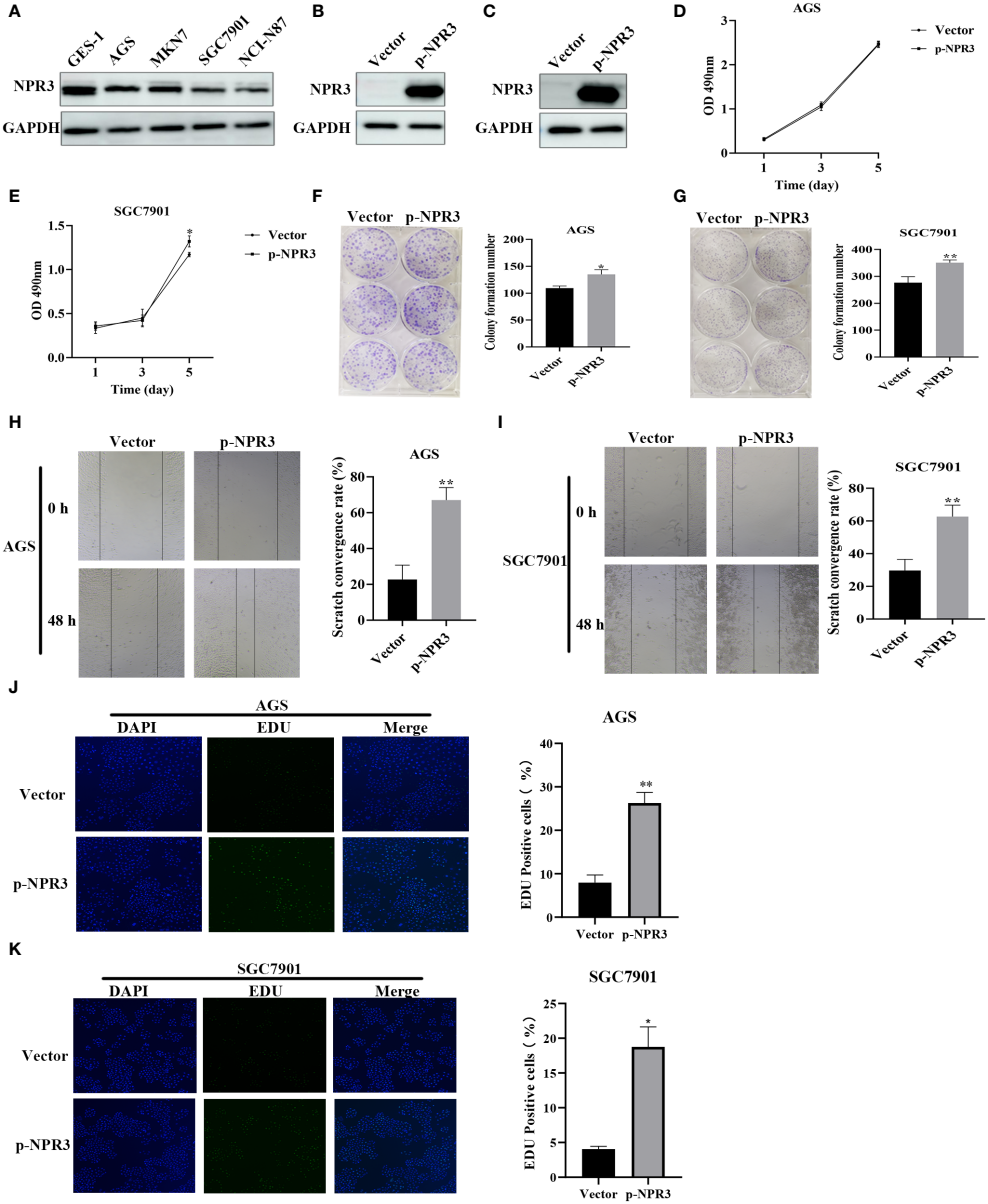
Function validation of *NPR3* in GC. **(A)** The expression of *NPR3* protein in GES-1, AGS, MKN7, SGC7901, NCI-N87 cells. **(B)** The expression of *NPR3* protein in AGS cells following transfection of p-NPR3. **(C)** The expression of *NPR3* protein in SGC7901 cells following transfection of p-NPR3. **(D)** Cell viability of AGS cells after transfection of p-NPR3. **(E)** Cell viability of SGC7901 cells after transfection of p-NPR3. **(F)** Clone formation of AGS cells after transfection of p-NPR3. **(G)** Clone formation of SGC7901 cells after transfection of p-NPR3. **(H)** Migration of AGS cells after transfection of p-NPR3. **(I)** Migration of SGC7901 cells after transfection of p-NPR3. **(J)** EdU assay of AGS cells after transfection of p-NPR3. **(K)** EdU assay of SGC7901 cells after transfection of p-NPR3. (*, ** represent P < 0.05, P < 0.01, respectively.).

The *NPR3* gene has been implicated in the pathogenesis of clear cell renal carcinoma (41), osteosarcoma (42), colorectal cancer (43), and hepatocellular carcinoma (44). Nevertheless, the potential role of *NPR3* in GC remains inadequately explored. Hence, we conducted additional investigations into the effect of *NPR3* on GC through cellular experiments. The western blot indicated a decreased expression of *NPR3* in GC cell lines (**Figure 9A**). The p-NPR3 was transferred into AGS and SGC7901 cell lines, and its efficiency for transfection was verified through a western blot (**Figures 9B, C**). AGS cell viability was not affected by *NPR3* overexpression (**Figure 9D**), while SGC7901 cell viability was significantly increased (**Figure 9E**). The colony formation experiments revealed that elevated levels of *NPR3* expression significantly promoted the formation of colonies in the SGC7901 and AGS cell lines (**Figures 9F, G**). Furthermore, migration experiments revealed that the upregulation of *NPR3* expression considerably improved the migratory ability of the SGC7901 and AGS cell lines (**Figures 9H, I**). According to the results of EdU assays, the proliferation of the SGC7901 and AGS cell lines was enhanced due to *NPR3* upregulation (**Figures 9J, K**). We further explored the effect of *NPR3* on epithelial-mesenchymal transition (EMT) in gastric cancer cells. The protein level of mesenchymal markers, such as N-cadherin, vimentin, were considerably increased while the expression of the epithelial marker E-cadherin was notably decreased in *NPR3* overexpressed cells (**Supplementary Figures S3A, B**). TIMER online database was used to analyze the correlation between *NPR3* and EMT-related genes. The findings demonstrated that *NPR3* was negatively correlated with E-cadherin (**Supplementary Figure S3C**) and positively correlated with N-cadherin, vimentin (**Supplementary Figures S3D, E**).

## 4 Discussion

GC, characterized by its heterogeneity, is frequently detected at an advanced stage (1). Although immunotherapy has emerged as a promising treatment strategy for GC (45, 46), its response rate remains suboptimal, presenting a substantial challenge in human health (1). Recent scientific investigations have highlighted the pivotal role of the cGAS-STING pathway in the body’s immune response to tumors (26, 29–31). Manganese, as the activator of this pathway (32–35), enhances the body’s anti-tumor capacity. The combination of manganese with ICIs has demonstrated improved antitumor effectiveness (36). These findings indicate that exploring manganese metabolism and immunity in GC has significant clinical implications for improving patient prognosis.

The progress achieved in bioinformatics and sequencing technologies has led to the development of several prognostic signatures associated with immunity, particularly in assessing the prognosis of individuals with GC (47–63). However, many prognostic models focus solely on immune-related genes, neglecting the impact of biological factors on the efficacy of immunotherapy. This study takes a novel approach by integrating research on immune and manganese metabolism, aiming to enhance the effectiveness of immunotherapy and deepen our understanding of the immunological characteristics of GC. KEGG and GO analysis revealed that DEGs associated with manganese metabolism and immune pathways were enriched in immune activation-related processes. Utilizing consensus clustering, we identified four distinct molecular subtypes, each exhibiting significant differences in survival rates. Further analysis explored the variations in tumor microenvironments, immune checkpoints, and enrichment pathways among these subtypes, confirming their impact on prognosis. Subsequently, a novel prognostic signature, termed MIRGs, was constructed, comprising 13 genes: *CD36, VCAN, SERPINE1, SLC24A2, RAG2, APOH, LGR6, CER1, VTN, NPR3, GRP, RNASE3*, and *EGF*. MIRGs effectively categorized GC patients into high-risk and low-risk groups, with the high-risk group demonstrating reduced overall survival time and poorer survival rates. Importantly, MIRGs were identified as independent predictors of the prognosis of GC patients. Nomograms were developed by combining various clinical variables with the risk score, providing a comprehensive perspective on the prognostic capabilities of MIRGs. This study, pioneering the integration of MRGs with IRGs, offers a unique and promising approach to prognostic modeling and suggests new avenues for therapeutic strategies in GC.

We further conducted a more in-depth investigation into the molecular mechanisms that contribute to the notable disparity in prognosis between distinct risk subgroups. The findings of immune cell infiltration analysis indicated a high proportion of monocytes and M2 macrophages in high-risk patients. Macrophages, which originate from monocytes, can be classified into two subtypes: M1 and M2 macrophages (64). Previous research has demonstrated that M2 macrophages are pivotal in the development and progression of GC. There is an increasing consensus (65, 66) suggesting that the infiltration of M2 macrophages is strongly linked to the immune evasion environment in GC. M2 macrophages typically release pro-angiogenic molecules, such as transforming growth factor-α and -β (TGF-α and -β), vascular endothelial growth factor (VEGF), which promote angiogenesis in GC (67, 68). M2 macrophages also facilitate the migration and infiltration of tumor cells by stimulating epithelial-mesenchymal transition (EMT) in GC (69–71). In addition, M2 macrophages induce the metastasis of gastric cancer by secreting CHI3L1 (72). Moreover, M2 macrophages have been observed to exacerbate the advancement of GC by modifying metabolism, specifically affecting fatty acids (73), arginine, proline (74), and methionine (75) metabolism. A high density of M2 macrophages has been identified as a predictive indicator of unfavorable outcomes in GC (76, 77). Consistently, a high abundance of M2 macrophages in patients at high risk for tumor progression and unfavorable outcomes was identified in our study. In addition to the above observation, numerous immune checkpoint expressions were substantially upregulated in individuals classified as high-risk. Immune checkpoints mediate co-inhibitory signaling pathways and induce tumor cells to evade immunosurveillance (78, 79). The combination of PD-1 expressed on immune cells and PD-L1 on antigen-presenting cells and tumor cells hinders the functioning of T cells and facilitates the evasion of the immune system by tumors (80). Besides, it is now understood that cytotoxic T-lymphocyte antigen-4 (CTLA-4) inhibits the binding of CD28 receptors on CD4^+^ T cells to B7 molecules on antigen-presenting cells. This activity impedes the transmission of signals from T-cell receptors, hence inhibiting their activation (81). T cell immunoreceptor with immunoglobulin and ITIM domain (TIGIT) contributes to suppress the immune response against malignancies through multiple routes. TIGIT suppresses dendritic cell maturation (82), inhibits natural killer cell effector function (83), and promotes macrophage polarization to the M2 phenotype (84). A study documented that lymphocyte activation gene-3 (LAG-3) can coordinate with PD-1 to promote the immune escape of GC, indicating its potential as an indicator of poor prognosis (85). The elevated levels of immune checkpoints were an additional factor contributing to unfavorable prognosis of high-risk patients. The high-risk group showed enrichment in pathways linked to cancer, which involves cell adhesion molecules, the TGF-β signaling pathway, and the JAK-STAT signaling system, as evaluated by GSVA. Cell adhesion molecules, particularly integrins, can enhance the survival, proliferation, and infiltration of tumor cells, hence facilitating the advancement and metastasis of malignancies (86). The TGF-β signaling pathway significantly influences the development of GC (87). Another study revealed a significant expression of TGF-β protein in GC, which contributes to the malignant transformation and proliferation of tumors (88). Additionally, TGF-β is widely recognized as the main inducer for the EMT pathway in GC (89). The JAK-STAT signaling system plays a role in the development and progression of GC. Previous research (90) demonstrated that the Janus kinase (JAK) dimers facilitate the phosphorylation of tyrosine 705 of signal transducer and activator of transcription 3 (STAT3), which is excessively active in GC. STAT3 governs the transcription of genes that facilitate tumor infiltration, cancer cell proliferation, and resistance to chemotherapy (91, 92). Besides, STAT3 promote mesothelial-to-mesenchymal transition and contributes to peritoneal metastasis of GC (93). The distinct molecular mechanisms may account for the poorer prognosis of individuals with GC who are classified as high-risk.

Herein, we conducted a comprehensive study on the significance of *NPR3* in GC. As a natriuretic peptide receptor, natriuretic peptide receptor 3 (*NPR3*) has been linked to the development of a variety of malignancies. Suppression of *NPR3* expression facilitated the spread of clear cell renal carcinoma (41). The induction of apoptosis in hepatocellular carcinoma cells was reported to result from the up-regulation of *NPR3* (44). The expression of *NPR3* inhibited the development of osteosarcoma by suppressing the PI3K-AKT pathway (42). In contrast, a study uncovered that *NPR3* overexpression promoted the proliferation of colorectal cancer cells (43). Moreover, *NPR3* was involved in constructing prognostic signatures to forecast the outcome of individuals with GC (60, 94) and breast cancer (95–97). It was found that *NPR3* actively stimulates the migration and proliferation of breast tumor cells (95). Nevertheless, the specific function of *NPR3* in gastric cancer has not been comprehensively examined. *NPR3* protein expression was demonstrated to be significantly downregulated in GC cell lines during experimental validation. The *NPR3* overexpression stimulated the migration and growth of GC cell lines, indicating that *NPR3* may function as a promoter of tumor growth in GC progression. Therefore, *NPR3* holds significant potential as a therapeutic and prognostic indicator for GC.

In summary, our research successfully developed a novel prognostic signature for gastric cancer incorporating 13 genes linked to manganese metabolism and the immune system. This prognostic signature exhibited outstanding predictive performance, providing valuable insights for clinical decision-making in GC treatment. The prospect of combining manganese with immune checkpoint inhibitors emerges as a promising avenue for future GC therapies. However, it is essential to acknowledge certain limitations in this research. The findings rely on publicly available databases and laboratory experiments conducted on isolated cells. Further investigations involving animal models and clinical trials are imperative before translating these findings into therapeutic applications.

## Data availability statement

The datasets presented in this study can be found in online repositories. The names of the repository/repositories and accession number(s) can be found in the article/**Supplementary Material**.

## Ethics statement

Ethical approval was not required for the studies on humans in accordance with the local legislation and institutional requirements because only commercially available established cell lines were used.

## Author contributions

XH: Conceptualization, Investigation, Methodology, Writing – original draft. CL: Conceptualization, Investigation, Methodology, Writing – original draft. SZ: Data curation, Formal analysis, Writing – review & editing. SSW: Data curation, Formal analysis, Writing – review & editing. SC: Investigation, Visualization, Writing – review & editing. SBW: Investigation, Visualization, Writing – review & editing. MZ: Investigation, Visualization, Writing – review & editing. XL: Validation, Visualization, Writing – review & editing. YL: Validation, Visualization, Writing – review & editing. BW: Project administration, Resources, Supervision, Writing – review & editing. WQ: Funding acquisition, Project administration, Supervision, Writing – review & editing.
