# Supplementary figures and images for: Development and verification of a manganese metabolism- and immune-related genes signature for prediction of prognosis and immune landscape in gastric cancer
Source: Front Immunol. 2024 May 13;15:1377472. doi: 10.3389/fimmu.2024.1377472 (PMC11131102; doi:10.3389/fimmu.2024.1377472)

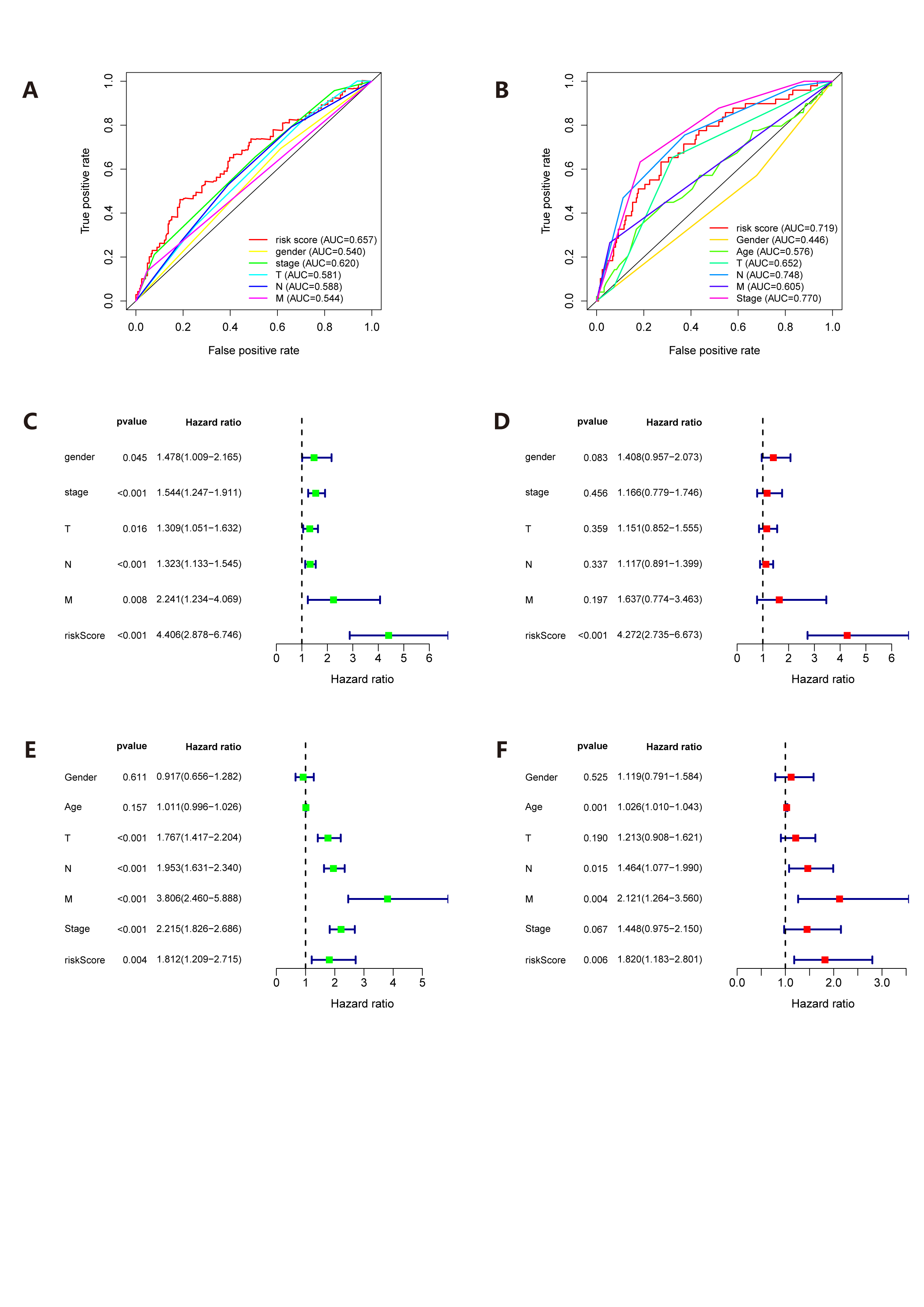

Supplement: Supplementary file 11 [file Image_1.tif]

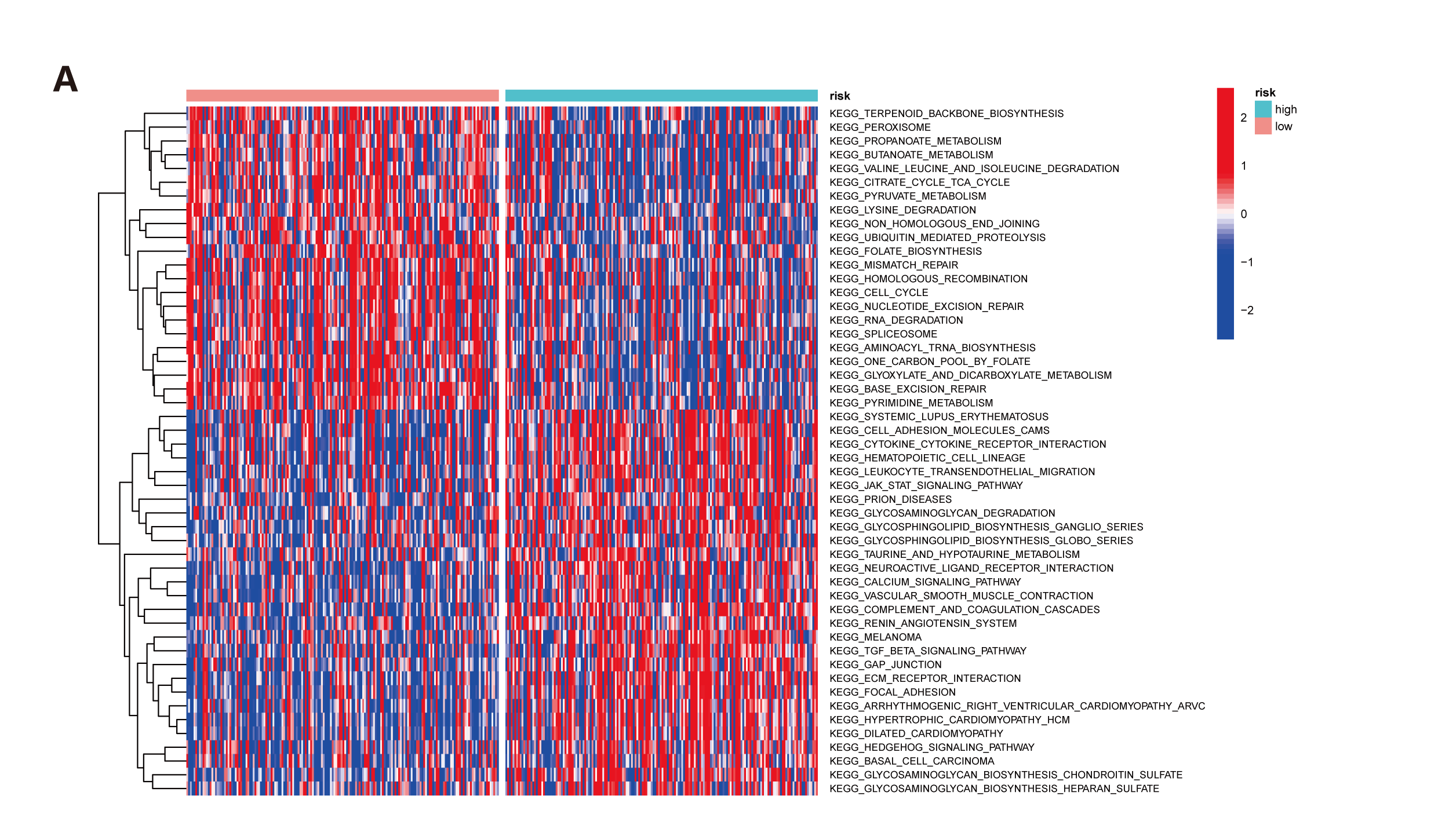

Supplement: Supplementary file 12 [file Image_2.tif]

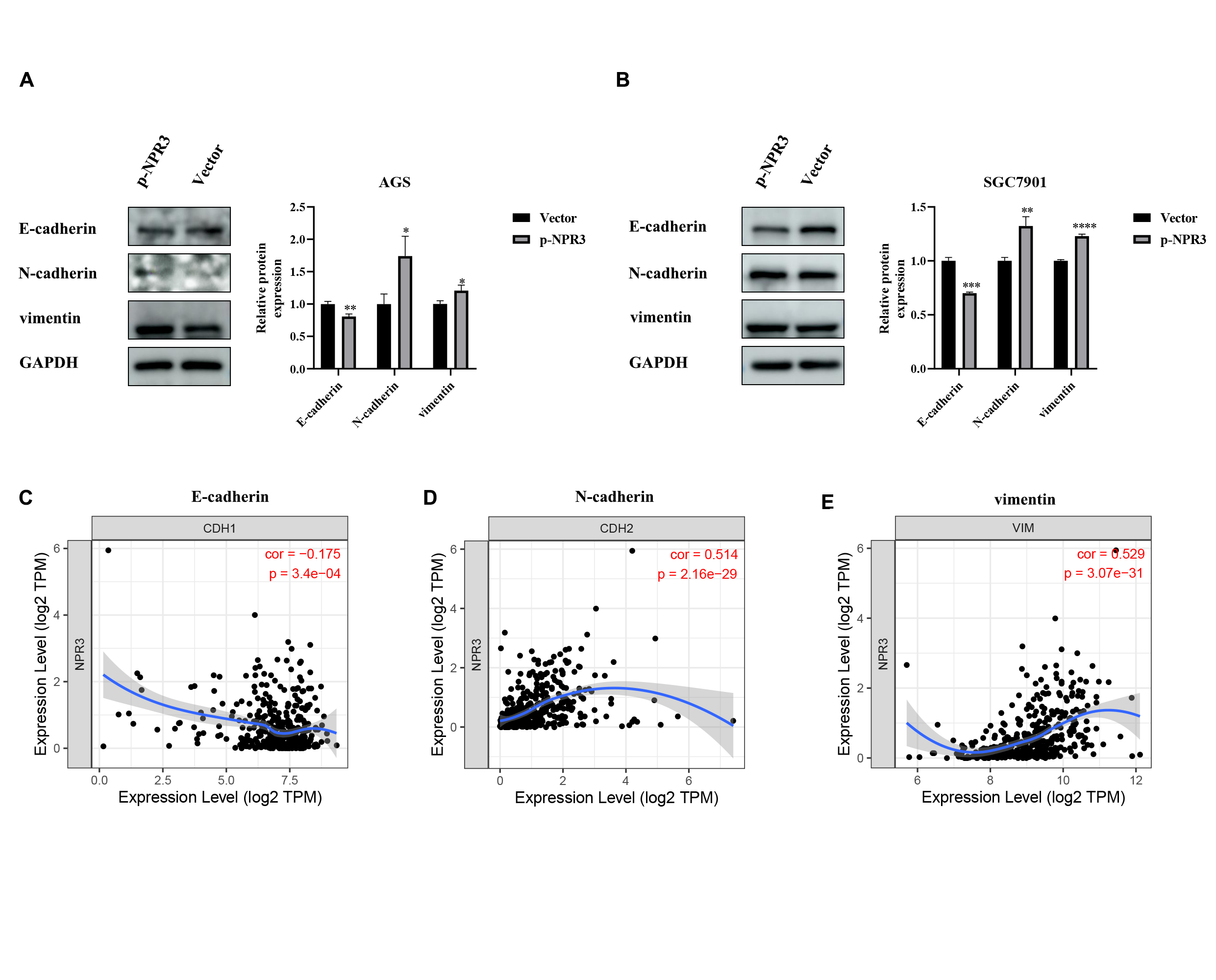

Supplement: Supplementary file 13 [file Image_3.tif]
